# Structural Plasticity of Intrinsically Disordered LEA Proteins from *Xerophyta schlechteri* Provides Protection *In Vitro* and *In Vivo*

**DOI:** 10.3389/fpls.2019.01272

**Published:** 2019-10-10

**Authors:** Mariana A. Silva Artur, Juriaan Rienstra, Timothy J. Dennis, Jill M. Farrant, Wilco Ligterink, Henk Hilhorst

**Affiliations:** ^1^Laboratory of Plant Physiology, Wageningen University, Wageningen, Netherlands; ^2^Department of Molecular and Cell Biology, University of Cape Town, Cape Town, South Africa

**Keywords:** intrinsic disorder, late embryogenesis abundant proteins, plant desiccation tolerance, resurrection plants, *Xerophyta*

## Abstract

Late embryogenesis abundant (LEA) proteins are essential to the ability of resurrection plants and orthodox seeds to protect the subcellular milieu against irreversible damage associated with desiccation. In this work, we investigated the structure and function of six LEA proteins expressed during desiccation in the monocot resurrection species *Xerophyta schlechteri* (XsLEAs). *In silico* analyses suggested that XsLEAs are hydrophilic proteins with variable intrinsically disordered protein (IDP) properties. Circular dichroism (CD) analysis indicated that these proteins are mostly unstructured in water but acquire secondary structure in hydrophobic solution, suggesting that structural dynamics may play a role in their function in the subcellular environment. The protective property of XsLEAs was demonstrated by their ability to preserve the activity of the enzyme lactate dehydrogenase (LDH) against desiccation, heat and oxidative stress, as well as growth of *Escherichia coli* upon exposure to osmotic and salt stress. Subcellular localization analysis indicated that XsLEA recombinant proteins are differentially distributed in the cytoplasm, membranes and nucleus of *Nicotiana benthamiana* leaves. Interestingly, a LEA_1 family protein (XsLEA1-8), showing the highest disorder-to-order propensity and protective ability *in vitro* and *in vivo*, was also able to enhance salt and drought stress tolerance in *Arabidopsis thaliana*. Together, our results suggest that the structural plasticity of XsLEAs is essential for their protective activity to avoid damage of various subcellular components caused by water deficit stress. XsLEA1-8 constitutes a potential model protein for engineering structural stability *in vitro* and improvement of water-deficit stress tolerance in plants.

## Introduction

Water availability is one of the major environmental factors that affect plant growth, development and productivity. During their life cycle, plants may endure periods of environmental drought and, depending on the duration of such periods, it may lead to irreversible structural damages affecting plant development and survival. Most higher plants undergo programmed water loss during their life cycle, which may occur in organs such as pollen, spores and seeds (Bewley, 1995; Scott, 2000). A group of about 135 angiosperm plant species have been described as “resurrection plants” for their ability to tolerate the loss of 80% to 95% of cellular water and resume photosynthetic activity and growth within hours after rehydration (Oliver et al., 2000; Scott, 2000; Porembski, 2011; Farrant et al., 2015). It is likely that desiccation tolerance (DT) appeared in the plant lineage during the transition from water to land, became confined to reproductive structures as plants evolved, and reappeared in vegetative structures of angiosperm resurrection plants by redirecting pre-existing genes and pathways to survive in the dry state (Ingram and Bartels, 1996; Oliver et al., 2000; Rensing et al., 2008; Farrant and Moore, 2011; Wodniok et al., 2011; Gaff and Oliver, 2013).

Recently, the genome of the resurrection species *Xerophyta schlechteri*^1^ (Behnke et al., 2013) has been sequenced (Costa et al., 2017). *X. schlechteri* is a monocot species belonging to the Velloziaceae family, and it is distributed mainly in southern African regions and inhabits rocky terrain or inselbergs in exposed grasslands (Porembski and Barthlott, 2000; Mello-Silva et al., 2011; Farrant et al., 2015). *X. schlechteri* is a resurrection monocot species, phylogenetically related to most important grass crops from the Poaceae family, and understanding its DT opens opportunities to apply this knowledge to improve drought tolerance in crops (Farrant et al., 2015; Costa et al., 2017).

Several mechanisms of responses to desiccation in *X. schlechteri* leaves have been correlated with its resurrection phenotype (Farrant et al., 2015). Among the essential adaptive mechanisms to survive loss of water, the accumulation of protective molecules has been shown to be an essential component of DT at the subcellular level. These molecules include sucrose, raffinose family oligosaccharides (RFOs) and LEA proteins which are thought to act, *inter alia*, as osmoprotectants in the formation and stability of the so-called cytoplasmic glassy state (Leprince et al., 1993; Hoekstra et al., 2001; Buitink and Leprince, 2004; Vicré et al., 2004; Farrant et al., 2012).

LEA proteins were first discovered due to their accumulation during late stages of embryo development of cotton seeds, coinciding with their acquisition of DT (Dure et al., 1981; Galau et al., 1986; Dure et al., 1989), and their characteristic stress-induced expression has led to the hypothesis that these proteins are involved in stress responses mediated by abscisic acid (ABA) (Galau et al., 1986). LEA transcription appears to be inducible by ABA and osmotic stress and is evident upon drying below 60% to 40% RWC, a common pattern observed in both seeds and resurrection plants (Illing et al., 2005; Leprince and Buitink, 2010; Costa et al., 2017). Interestingly, the translation of some LEA proteins has been shown to occur a few hours or even days after their transcription, suggesting regulation at the transcriptional and translational levels (Galau et al., 1987; Hughes and Galau, 1991; Espelund et al., 1992; Chatelain et al., 2012; Verdier et al., 2013). In addition to transcriptional and translational regulation, some LEA proteins were shown to undergo posttranslational modifications (PTMs), reflecting the complexity of the regulation of these proteins at various levels (Riera et al., 2004; Boudet et al., 2006).

Several LEA proteins are assumed to be intrinsically disordered proteins (IDPs) considering their ability to undergo order to disorder transitions in different *in vitro* environments (Soulages et al., 2002; Mouillon et al., 2006; Shih et al., 2010; Popova et al., 2011; Hincha and Thalhammer, 2012; Hundertmark et al., 2012; Shih et al., 2012; Rivera-Najera et al., 2014; Cuevas-Velazquez et al., 2016; Bremer et al., 2017; Cuevas-Velazquez et al., 2017). This interesting physicochemical characteristic allows LEA proteins to form homo- and heterodimers and to interact with multiple targets (Tolleter et al., 2007; Popova et al., 2015; Cuevas-Velazquez et al., 2016; Hernandez-Sanchez et al., 2017). The ubiquitous distribution of LEA-like proteins in bacteria and invertebrates suggests that similar protective mechanisms of DT involving LEA proteins have evolved across different life forms (Tunnacliffe and Wise, 2007; Costa et al., 2016).

The myriad of secondary structures enables LEA proteins to play multiple roles in abiotic stress tolerance, constituting an essential footprint of DT in seeds and resurrection plants (Maia et al., 2011; Costa et al., 2017). To further characterize and explore the biochemical, structural, and functional properties of LEA proteins, we cloned the coding sequences of six *LEA* genes expressed in *X. schlechteri* leaves upon desiccation: *XsDHN12*, *XsLEA1-8, XsLEA4-8*, *XsLEA4-12, XsLEA6-2*, and *XsSMP4*. We examined the secondary structure of these LEA proteins by circular dichroism (CD) and monitored their folding dynamics in hydrophobic solution. *In vitro* and *in vivo* experiments demonstrated that these XsLEAs perform protective roles under desiccation, heat, oxidative, salt, and osmotic stress. The protective activity of XsLEAs seems protein type-specific, with a strong correlation between the ability to form defined secondary structures *in vitro* and the extent of protection both *in vitro* and *in vivo*. XsLEAs are localized in multiple subcellular compartments, such as nucleus, membrane, and cytoplasm, supporting the idea of universal cell protection by LEA proteins. Heterologous expression of *XsLEA1-8* in *Arabidopsis thaliana* leads to higher tolerance to salt and osmotic stress in seedlings and drought in adult plants. Our work sheds new light on the biochemical properties of these stress-responsive proteins and highlights characteristics of LEA proteins that can be useful for bioengineering protein stability *in vitro* and improving abiotic stress tolerance in crops.

## Materials and Methods

### *In Silico* Analysis

Protein Grand Average Hydropathy was calculated using the GRAVY calculator (http://www.gravy-calculator.de/). Molecular mass and isoelectric point (pI) were calculated with the isoelectric point calculator (IPC) (Kozlowski, 2016). Protparam (http://web.expasy.org/protparam/) was used to analyse amino acid composition and predict protein stability. The percentage of polar residues was calculated with EMBOSS PEPSTATS (https://www.ebi.ac.uk/Tools/seqstats/emboss_pepstats/). Prediction of the degree of protein disorder was performed using IUpred (Dosztányi et al., 2005) and PONDR (http://www.pondr.com/) with default parameters (VLXT predictor). Sequence-specific parameters and prediction of structural qualities of the proteins were predicted with CIDER (http://pappulab.wustl.edu/CIDER/analysis/) (Holehouse et al., 2017). ANCHOR web server (http://anchor.enzim.hu/) was used to predict the number of disordered binding regions (DBRs), i.e. regions with propensity to undergo folding upon partner-binding with a probability higher than 50%, and MoRFpred (http://biomine.cs.vcu.edu/servers/MoRFpred/) was used to predict the number of residues of molecular recognition features (MoRFs). Disorder enhanced phosphorylation predictor (DEPP), also known as DisPhos and available from http://www.pondr.com/cgi-bin/depp.cgi was used to predict phosphorylation sites within the protein sequences (Iakoucheva et al., 2004). Subcellular localization was predicted with PSI (http://bis.zju.edu.cn/psi/), CELLO2GO (http://cello.life.nctu.edu.tw/) and Plant-mPLoc (http://www.csbio.sjtu.edu.cn/bioinf/plant-multi/). Signal peptide predictions were performed with SignalP v. 4.1 Server (http://www.cbs.dtu.dk/services/SignalP/), PrediSi (http://www.predisi.de/), and TargetP 1.1 Server (http://www.cbs.dtu.dk/services/TargetP/). 

### Plant Materials and Growth Conditions

Seeds obtained from mature *X. schlechteri* plants, collected from Buffelskloof Private Nature Reserve in the Mpumulanga province of South Africa, were sown on potting soil and maintained in greenhouse at the Wageningen University under conditions of 16 h at 27°C during the day and 8 h at 18°C during the night. Dehydration was achieved by withholding water from the pots. Young green leaf tissue was harvested from four individual plants on a daily basis, frozen in liquid nitrogen, and stored at −80°C. *Nicotiana benthamiana* plants used for agro infiltration and *A. thaliana* plants ecotype Columbia-0 used for floral dipping and drought treatments were grown on a mix of 50% vermiculite and 50% soil watered three times a week with Hyponex solution (Hyponex Japan, Osaka, Japan http://www.hyponex.co.jp), in a greenhouse with long-day photoperiod cycles (16 h light/8 h dark) at 22°C ± 2°C for 3 weeks. *A. thaliana* plants used for seed collection were grown on Rockwool blocks (Grodan, the Netherlands) in Hyponex solution under greenhouse conditions (16 h light/8 h dark).

### RNA Extraction, cDNA Synthesis and Cloning of *XsLEAs*

Total RNA was extracted from leaves of adult *X. schlechteri *plants 9 days after dehydration using the hot borate protocol (Wan and Wilkins, 1994). cDNA was synthesized using the iScript^™^ cDNA Synthesis Kit (Bio‐Rad, Laboratories B.V., The Netherlands) according to the manufacturer’s protocol. Primers were designed to include flanking restriction sites, which were in turn flanked with Gateway AttB1 and AttB2 sites (**Supplementary table 1**). PCR was performed on *X. schlechteri* cDNA with Q5 high-fidelity DNA polymerase (New England Biolabs) and PCR products were purified from gel using Nucleospin Gel and PCR clean-up (Macherey-Nagel). The purified amplicons were cloned into the entry vector pDONr201 using BP Clonase II according to the manufacturer’s instructions (Thermo Fisher Scientific) to create pENTr201-LEA and sequenced to confirm the gene sequence.

### Construction of Plant Expression Vectors

For *N. benthamiana* transient gene expression, subcloning from pENTr201-LEA into pGWB606 (Nakamura et al., 2010) was performed with a Gateway LR reaction to produce recombinant *p35S::GFP-XsLEAs*. Correct reading frame was confirmed by sequencing. For *A. thaliana* heterologous expression, subcloning of the *XsLEAs* CDS from pENTr201-LEA into the expression vector pB7WG2RS was also performed with a Gateway LR reaction. The expression vector pB7WG2RS was made by cloning the RedSeed selection marker (*pNAP::DsRed*) from pKGW-RedSeed between the left T-DNA border and the *Bar* resistance gene of pB7WG2 (Karimi et al., 2002) using XbaI and KpnI. Correct insertion of the RedSeed marker was confirmed by sequencing.

### Construction of Bacterial Expression Vectors

For protein expression and purification, *XsLEA* CDSs were cloned from pENTr201-LEA into bacterial expression vector pDEST17 with a Gateway LR reaction to produce recombinant N-terminal His-tagged proteins (pEXPR17-LEA). The correct reading frame was checked by sequencing. For *E. coli* stress assays, the *XsLEAs* were subcloned from pENTr201-LEA to the bacterial expression vector pCDF-Duet (Novagen), which contains two multiple cloning sites (MCSs), using traditional cloning. *XsLEA6-2* and *XsDHN12* were subcloned to MCS1 using NcoI and EcoRI, while *XsSMP4*, *XsLEA4-8*, *XsLEA4-12*, and *XsLEA1-8* were subcloned to MSC2 using NdeI and AvrII (New England Biolabs; **Supplementary Table 1**). In all cases, both pENTr201-LEA and pCDF-Duet were digested with the corresponding enzymes. The fragment containing the *XsLEA* CDS from the digested pENTr201-LEA was purified by gel extraction (Macherey-Nagel). The digested pCDF-Duet vector was subjected to dephosphorylation (shrimp alkaline phosphatase [rSAP] from New England Biolabs) and purified with a silica column (Macherey-Nagel). The *XsLEA* fragments were ligated into pCDF-Duet using T4 DNA Ligase (Promega) to create pCDF-LEA. Correct orientation was confirmed by PCR.

### Expression and Purification of the Recombinant XsLEAs

BL21 (DE3) pLysS (Novagen) *E. coli* expression strain carrying the different pDEST17-LEA vectors were grown overnight in 5-cm^3^ sterile LB medium at 37°C (200 rpm). The cultures were inoculated in 1 dm^3^ of sterile LB medium in a 5-dm^3^ Erlenmeyer flask and incubated at 37°C with shaking (200 rpm). When an optical density at 600 nm (OD_600_) of 0.6 was reached (± 3 h), isopropyl β-d-thiogalactopyranoside (IPTG) was added to a final concentration of 1 mM and incubation continued for 2 h at 37°C (100 rpm) to induce protein expression. The cells were then harvested by centrifugation at 4°C, 10,000*g* for 10 min and the cell pellets were frozen overnight at −20°C. The cell pellets were then thawed an resuspended in 15 cm^3^ Lysis-Equilibration-Wash Buffer (LEW; Macherey-Nagel) containing 50 mM NaH_2_PO_4_ and 300 mM NaCl with a pH of 8.0 using NaOH. A 1-mg/cm^3^ lysozyme (Sigma-Aldrich) was added, followed by incubation on ice for 30 min. The cells were lysed by sonication and the lysate was centrifuged for 30 min at 4°C, 10,000*g*. For purification of the His-tagged recombinant LEA proteins from the crude lysate, the Protino^®^ Nickel TED (Ni-TED) Histidine Tag Affinity Purification Kit (Macherey-Nagel, Germany) was used with standard protocol. The purified His-tagged XsLEAs were subjected to concentration and buffer exchange into dH_2_O using the Amicon Ultra Centrifugal Filters (3K for XsLEA4-8, XsLEA1-8, XsLEA6-2 and XsDHN12, and 30K for XsSMP4 and XsLEA4-12, MWCO, Merck Millipore). Centrifugation was performed at 8,000*g* for 1 h or 14,000*g* for 15 min for the 3K and 30K filters, respectively, at 4°C to concentrate the proteins, followed by three washing steps with 12 cm^3^ of dH_2_O (the same centrifugation parameters were used). Total protein concentration was quantified using the Bradford BioRad Microassay (BioRad USA) according to manufacturer’s instructions with bovine serum albumin (BSA; Sigma Aldrich, USA) as a standard. As an additional purification step, making use of the heat stability of most LEA proteins (Boudet et al., 2006), 10 to 15 µg of the recombinant proteins were submitted to a 97°C treatment for 10 min, centrifuged, and the supernatant was analyzed on a 12% SDS-PAGE at 90 V for 2 h with a Colour Prestained Protein Standard Ladder (New England Biolabs, USA). After electrophoresis, gels were stained with a Coomassie Blue solution (2.5 g/dm^3^ Coomassie Blue (Sigma-Aldrich), 50% v/v methanol, 10% v/v acetic acid) for 1 h at room temperature and destained overnight in a solution of 45% v/v methanol and 10% v/v acetic acid prior to visualization.

### In-Solution Circular Dichroism Analysis for Secondary Structure

Circular dichroism (CD) spectra of the six recombinant His-tagged LEA proteins were obtained using a JASCO J-810 Spectropolarimeter (JASCO Analytical Instruments, Japan) in 1 mm path-length quartz cuvettes. Spectral data were recorded from 240 to 190 nm, with 10 accumulations per run using a 0.2-nm data pitch, 100 nm/min scanning speed, 1 s response time and 1 nm of band width. To simulate changes in secondary structure, we used 80% acetonitrile (ACN), 20 mM NaCl, and MiliQ water adjusted with HCl to pH 2.3 or pH 4.0 as protein solvents. BSA (Sigma-Aldrich, USA) was used as a control for the spectra of an alpha-helical structure, at a concentration of 0.075 mg/cm^3^. Measurements of millidegrees obtained from the results were subsequently converted into mean residue (θ) and plotted against the wavelength range (nm). CD data were fitted using Dichroweb (http://dichroweb.cryst.bbk.ac.uk/html/home.shtml) (Contin, data set 7) (Whitmore and Wallace, 2004) to estimate the secondary structure content.

### *In Vitro* Lactate Dehydrogenase Assays

Lactate dehydrogenase (LDH) assays were adapted from Reyes et al. (2008). In short, LDH from rabbit muscle (Sigma-Aldrich) was diluted to a final concentration of 200 nM in 25 mM Tris pH 7.5. The purified XsLEAs or BSA were also diluted to a final concentration of 200 nM in 25 mM Tris-HCl pH 7.5. The mixture of LDH and XsLEA at a molar ratio of 1:1 was submitted to desiccation, heat or oxidative stress. After each treatment, the enzyme and protein mixture was added to a reaction buffer containing 25 mM Tris-HCl pH 7.5, 2 mM of pyruvate (Sigma-Aldrich) and 0.15 mM NADH (Roche) to a final volume of 1 cm^3^ in 2 cm^3^ plastic cuvettes and the initial absorbance was measured at 340 nm. The rate of the decrease in absorbance due to the conversion of NADH to NAD^+^ was determined every 5 s for 1.5 min at 25°C. For the desiccation-induced aggregation assay, LDH in the presence of each of the purified XsLEAs or BSA were submitted to dehydration in a centrifugal evaporator (Savant™ SpeedVac™ Plus SC210A) for 1 h at room temperature. After dehydration, the initial volume was restored by adding 25 mM Tris-HCl pH 7.5, and enzyme activity was measured as mentioned above. For the thermal inactivation assays, the samples containing each of the six purified XsLEAs or BSA together with LDH were heated at 42°C for 20 min and cooled down at room temperature for 10 min. Oxidative stress was imposed by incubating the LDH enzyme and XsLEAs or BSA mixtures in 200 mM of H_2_O_2_ (Sigma-Aldrich) at room temperature for 1 h. Each assay was repeated three times with three technical replicates each and statistically significant differences were analyzed with Excel (Microsoft, United States) using Student’s *t*-test.

### Abiotic Stress Tolerance Assays of *E. coli* Transformants

*E. coli* strain BL21(DE3)RIL (Agilent) was transformed with the pCDF-Duet-LEA vectors. LEA expression was induced with 1 mM IPTG for 2 h. Cells were diluted to OD_600_ = 1.0 and a serial dilution of 5 mm^3^ was spotted onto LB media containing 350 mM of NaCl or mannitol. To assess *in vivo* heat protective function 1 cm^3^ of LB containing IPTG-induced *E. coli* cells (OD_600_ = 1.0) was incubated in a water bath at 50°C for 30 min, cooled for 10 min at room temperature and then spotted onto LB control plates supplemented with 1.5% Daishin agar (Duchefa). Serial dilutions at 10, 50, 100, 500, and 1000 times were used for salt and osmotic stresses, and 0, 10, 100 and 1000 times for heat stress assays. The plates were incubated at 37°C for 16 h. For the liquid media growth assay, 1 cm^3^ of cells at OD_600_ = 1.0 was diluted 10 times with LB liquid media supplemented with 250 mM NaCl. The cells were kept at 37°C and 100 mm^3^ aliquots were taken every hour for measuring the OD_600_. Due to the possibility of leaky vector activation, resulting in expression of proteins before induction with IPTG (Grossman et al., 1998; Zhang et al., 2015; Briand et al., 2016), we analyzed and compared the relative growth percentage of the XsLEA strains with the empty vector-carrying strain. The difference in OD_600_ at time point *x* (t*_x_*) between treated and non-treated (control) cultures were used to calculate the relative growth, expressed as [OD_treatment(t_ *_x_*_)/_OD_control(t_ *_x_*_)_] × 100. Each experiment was repeated twice and statistical differences were determined by using the Student’s *t*-test in Excel (Microsoft, United States).

### *In Vivo* Localization of XsLEAs In *N. benthamiana* Leaves

*Agrobacterium tumefaciens* strain AGL0, carrying constructs of pGWB606 *p35S::GFP-LEAs*, was grown in LB medium containing antibiotics and harvested by centrifugation at 4,000*g* for 5 min at room temperature. The bacteria culture was resuspended in infiltration buffer (10 mM MgCl_2_, 10 mM MES, pH 5.6, and 200 μM acetosyringone) and adjusted to OD_600_ = 0.6. The bacterial suspension was incubated at room temperature on a rocking platform for 1 h. Leaves of 3- to 4-week-old *N. benthamiana* plants were infiltrated with *A. tumefaciens* suspension using a needleless syringe and a minimum of three independent agro infiltrations was performed (*n* ≥ 3). Three days after infiltration, two leaves from three independently transformed plants were analyzed for GFP fluorescence under a Leica TCS SP8 HyD confocal microscope (Leica) with an excitation wavelength of 488 nm, and the spectral detection was set between 500 and 557 nm for GFP, and 642 and 747 nm for chlorophyll fluorescence. The objective used was 40× in water immersion.

### *A. thaliana* Transformation and RT-qPCR Gene Expression Confirmation

The pB7WG2RS *p35S::LEA1-8* construct was introduced into *A. tumefaciens* strain AGL0 and transformed into *A. thaliana* Colombia-0 (Col-0) using the floral dip method (Clough and Bent, 1998). Using the RedSeed marker, transgenic T1 plants were selected and single T-DNA inserts were identified in the T2. RNA of T3 dry seeds was extracted and 700 ng of RNA was reverse transcribed as described above. RT‐qPCR reactions were run on a CFX machine (Bio‐Rad). Three technical replicates were used per sample. The reference genes used for data normalization were At4g12590 and At4g34270 (Dekkers et al., 2012). The primers used for RT-qPCR and the obtained C_t_ values are presented in **Supplementary Table 2**. After confirmation of the expression of *XsLEA1-8* in T3 seeds, plants were grown in a complete randomized design containing three biological replicates of at least four plants. Seeds were harvested and used for further experiments.

### Seed, Seedling, And Plant Stress Phenotyping

Germination experiments were performed 10 days after harvest. For control conditions, seeds were sown in square trays on two layers of filter papers saturated with dH_2_O, in accordance with Joosen et al. (2010). For salt and osmotic stress, the filter papers were saturated with −0.3 MPa NaCl or −0.6 MPa mannitol, respectively. After sowing, the seeds were stratified at 4°C in the dark for 48 h and the trays were subsequently moved to 22°C under continuous light. To investigate tolerance to deterioration conditions, dry seeds were incubated at 40°C at approximately 85% relative humidity for 3 days, and then transferred to germination conditions at 22°C under continuous light. Germination was scored twice a day for 5 days using the Germinator program (Joosen et al., 2010). For seedling stress assays, seeds were stratified at 4°C in the dark for 72 h on square 14-cm Petri dishes on ½ Murashige-Skoog (MS) medium supplemented with 0.5% sucrose, 0.1% MES monohydrate and 1% Daishin agar, pH 5.8 (KOH). After 4 days, seedlings were transferred to the same medium supplemented with 100 mM NaCl or 200 mM Mannitol. Each plate contained four seedlings of three genotypes, and three biological replicates per genotype were used following a complete randomized design. The plates were vertically placed at 21°C under continuous light. The primary root length was scored after 10 days. Drought stress was assessed by withholding water from 3-week-old *A. thaliana* plants grown on soil in the greenhouse. After 12 days, the percentage water content from the leaves was measured, and plants were rewatered. Final survival was analyzed 7 days after rewatering. Three biological replicates with three plants were used per time point. Leaves and soil fresh weight (FW) were measured immediately after harvest, and the dry weight (DW) was measured after 48 h at 60°C. Percentage water content (%) from the plants and soil was calculated as (FW − DW)/FW × 100.

## Results

### XsLEAs Are Predicted To Be Intrinsically Disordered

The expression of the six *XsLEAs* used in our analysis has been reported by Costa et al. (2017) to be increased in *X. schlechteri* leaves upon desiccation between 60% RWC (1.5 gH_2_O g^−1^ dwt) and 40% RWC (1.0 gH_2_O g^−1^ dwt) (**Supplementary Figure 1**), coinciding with the activation of the molecular signature of the resurrection physiology. In parallel, genes related to protein folding, protection and translational control were also enriched.

The cDNAs of the *XsLEAs* contained an open reading frame (CDS) ranging from 309 to 1149 nucleotides encoding proteins with 102 to 382 amino acids with molecular weights between 10.73 and 38.92 kDa (**Table 1**). The theoretical pI ranged from 4.3 to 8.6, indicating that *XsLEAs* expressed upon desiccation constitute acidic, neutral, and basic proteins. All six XsLEAs had a negative GRAVY index, a common characteristic of hydrophilic LEA proteins (Battaglia et al., 2008).

**Table 1:** Characteristics of *Xerophyta schlechteri* late embryogenesis abundant (LEA) proteins.

Gene ID*	PFAM	Name	CDS size	Number of amino acids	Molecular weight (kDA)	Theoretical pI	GRAVY
Xvis02_06457	pf04927	XsSMP4	918	305	31.68	4.3	−0.437
Xvis02_11331	pf02987	XsLEA4-8	441	146	15.55	6.6	−1.316
Xvis02_12059	pf02987	XsLEA4-12	1149	382	38.92	5.9	−0.872
Xvis02_20008	pf03760	XsLEA1-8	321	106	10.73	8.6	−0.916
Xvis02_08790	pf10714	XsLEA6-2	309	102	11.11	5.0	−1.095
Xvis02_23545	pf00257	XsDHN12	372	123	13.44	7.2	−1.389

*Gene IDs were retrieved from (Costa et al., 2017).

The percentage of polar residues was relatively higher compared with non-polar residues in all XsLEAs, with the exception of XsSMP4 with 52.79% non-polar residues (**Supplementary Table 3**). The disorder promoting amino acids alanine (A), lysine (K), glycine (G), glutamine (Q), and glutamic acid (E) were enriched in at least four of the six sequences. These characteristics indicate that the majority of the studied XsLEAs are likely to be IDPs (Dunker et al., 2001; Tompa, 2002). To verify the IDP properties of the XsLEAs, several *in silico* analyses were performed. All XsLEAs were predicted to belong to the category of proteins with extended disorderedness (**Supplementary Figure 2A**). With the exception of XsSMP4, all XsLEA protein sequences displayed high disorder tendency (**Supplementary Figure 2B**). XsSMP4 appeared to be the least disordered from the proteins herein investigated, because it displayed higher hydropathy and GRAVY (**Table 1**), as well as higher percentage of non-polar residues (**Supplementary Table 3**).

*In silico* structural analysis indicated that four proteins (XsLEA1-8, XsLEA4-8, XsLEA6-2, and XsDHN12) are predicted as Janus sequences, which are proteins that undergo environment-dependent conformational transitions (**Supplementary Table 4**). XsSMP4 is predicted to form tadpole and globular structures, in agreement with the less disordered nature of this protein. XsLEA4-12 is predicted to form coils, hairpins, and chimeras, representing a structurally more heterogeneous protein type. It is important to highlight that despite *in silico* analysis that allows the prediction of structural propensities in IDPs, these proteins exist as ensembles of conformations in natural conditions, which can be a balance between more ordered and disordered structures (Das et al., 2015).

DBRs are common among IDPs and may contain short fragments of about 5 to 25 residues named MoRFs that are prone to undergo disorder-to-order transitions in the presence of binding partners (Mohan et al., 2006). We found that the number of DBRs as well as the number of MoRFs was variable among the six XsLEAs and that the position of MoRFs not always correlated with those of DBRs (**Supplementary Table 4**, **Supplementary Figure 3**). XsDHN12 and XsLEA1-8 presented longer MoRFs within DBRs in the C-terminus, and XsLEA6-2 presented two longer MoRFs within DBRs located in the N- and C-terminus. Interestingly, the three proteins presenting larger number of smaller DBRs (XsLEA4-8, XsLEA4-12, and XsSMP4), also presented no or smaller MoRFs within DBRs (**Supplementary Figure 3**). These observations indicate that there is a high variability regarding the number, size, and locations of DBRs and MoRFs between the different XsLEA proteins and suggest that XsDHN12, XsLEA1-8, and XsLEA6-2 may undergo higher conformational changes in the presence of binding partners.

The binding affinity of MoRFs is believed to be modulated by phosphorylation of MoRF residues (Mohan et al., 2006). We investigated the percentage and location of phosphorylation residues (Ser, Thr, and Tyr) of the six XsLEAs (**Supplementary Figure 3**, **Supplementary Table 4**). The percentage of predicted phosphorylation sites was variable between the six LEA proteins, with XsLEA4-12 showing the highest percentage (20.7%) followed by XsLEA4-8 (13.2%). Interestingly, in most of the proteins analyzed, the predicted phosphorylation sites were located outside the MoRF regions (**Supplementary Figure 3**) suggesting that phosphorylation may not be the major modulator of binding affinity in XsLEAs *via* MoRF recognition, although further experimental data are still needed to confirm this *in silico* observation.

### XsLEAs Are Intrinsically Disordered in Aqueous Solution and Display Conformational Plasticity

His-tagged recombinant XsLEAs were produced to investigate their structure in solution. Similar to other LEA proteins (Kovacs et al., 2008; Hundertmark et al., 2011), all the XsLEAs showed slightly higher molecular masses in an SDS gel than the predicted mass (**Table 1**, **Supplementary Figure 4**). This lower gel mobility might be due to the 6xHis-tag or due to the hydrophilic character of IDPs (Tompa, 2002; Kovacs et al., 2008). All XsLEAs used in our analysis were confirmed to be heat stable, a common characteristic of LEA proteins, which are known to be a part of the heat-stable proteome associated with DT in seeds (Boudet et al., 2006; Kovacs et al., 2008; Chatelain et al., 2012).

We investigated the disordered nature and folding behavior of the six XsLEAs in aqueous solution by CD spectroscopy. BSA was used as a positive control protein due to its predominantly α-helical conformation (Reed et al., 1975). This is reflected in the BSA spectra, which characterized by a positive maximum at ∼190 nm and negative minimum at 208 nm and 220 nm (**Supplementary Figure 5A**). In general, the spectra of the six analyzed XsLEAs were characterized by a negative minimum between 198 and 201 nm in water (**Supplementary Figures 5B–G**), which is characteristic of natively unfolded proteins (Uversky, 2009; Lopes et al., 2014). A residual alpha-helix content was commonly observed in all XsLEAs in aqueous solution, and the presence of turns or strands was variable between the six proteins (**Supplementary Figure 5H**). Taken together, these results confirm that the six investigated XsLEAs are proteins with intrinsically disordered regions (IDRs).

**Figure 1:**
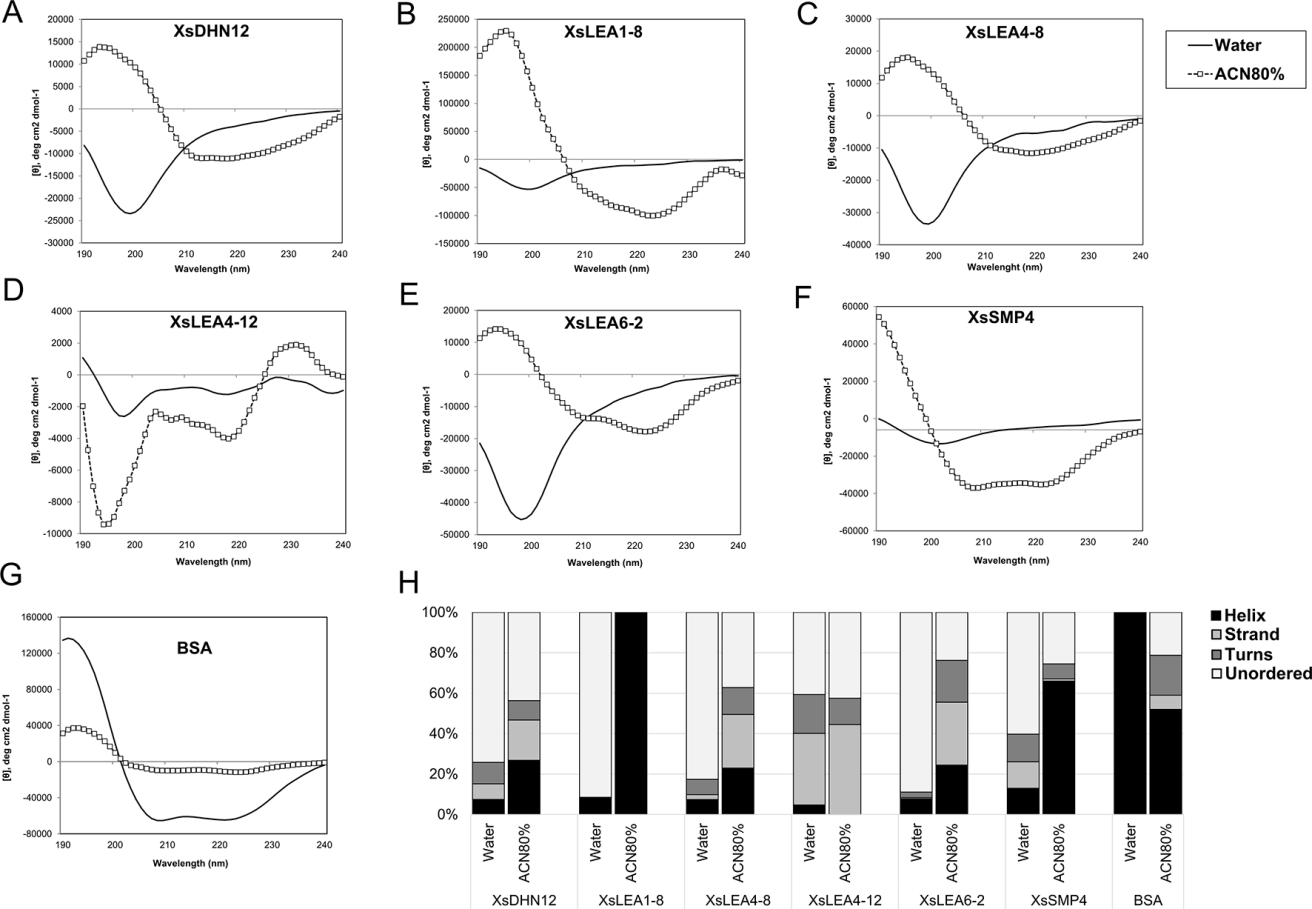
Normalized CD spectra of *Xerophyta schlechteri* late embryogenesis abundant (LEA) proteins and bovine serum albumin (BSA). The CD spectra were obtained in water and 80% acetonitrile (ACN). All the spectra were analyzed at room temperature **(A-G)**. The graphs show the spectra obtained after subtracting the reads of a blank sample containing water only. **(H)** Secondary structure content of *X. schlechteri LEAs* (XsLEAs). Analyses of the CD data to obtain an estimation of the content of helix, strand, turns and unordered conformations were performed with Dichroweb.

To test the effect of changes in the pH and composition of the solvent we analyzed the spectra of the six XsLEAs in aqueous solutions adjusted with HCl to pH 4.0 or pH 2.3, and in the presence of 20 mM NaCl or 80% ACN. The decrease in the pH or addition of 20 mM of NaCl did not significantly affect the spectra of BSA when compared to aqueous solution and had little effect on the secondary structure content of XsLEAs (**Supplementary Figures 5A–H**). The most noticeable secondary structure variations in these conditions were observed for XsLEA1-8, with an increase in helix content at pH 2.3, and for XsLEA4-8, with an increased strand formation in NaCl solution (**Supplementary Figure 5H**). On the other hand, high concentrations of the organic solvent ACN resulted in considerable changes in the secondary structure of XsLEAs (**Figures 1A–F**). ACN is an organic water miscible solvent that can increase protein chemical potential leading to conformational changes, acting as a denaturant, as well as an alpha-helix-promoting agent (Gekko et al., 1998). With the exception of XsLEA4-12, a decrease of the negative minima (between 197 and 200 nm) converting it to a positive signal and an increased negative signal between 210 and 240 nm was commonly observed in the spectra of all XsLEAs in 80% ACN solution, resulting in a reduction of unordered regions (**Figure 1H**).

The protein XsLEA4-12 showed atypical behavior in 80% ACN, with increased negative signal around 195 and 200 nm, and a positive signal near 230 nm. The spectrum of this protein between 195 and 220 nm resembles that of a denatured or aggregated protein (Venyaminov et al., 1993; Greenfield, 2006). Different from what was observed for XsLEAs, BSA showed a decrease in helix content and appearance of disordered, turns and strand conformations in 80% ACN (**Figures 1G, H**). Interestingly, XsLEA1-8 underwent a conformational change from about 92% disordered in aqueous solution up to 100% alpha-helix in 80% ACN (**Figure 1H**). In summary, our results suggest that XsDHN12, XsLEA1-8, XsLEA4-8, XsLEA6-2, and XsSMP4 display conformational plasticity and underscore the plasticity of XsLEA1-8 to acquire high degrees of secondary structure in a highly hydrophobic environment.

### XsLEAs Stabilize Enzyme Activity Upon Stress in a Protein-Specific Manner

**Figure 2:**
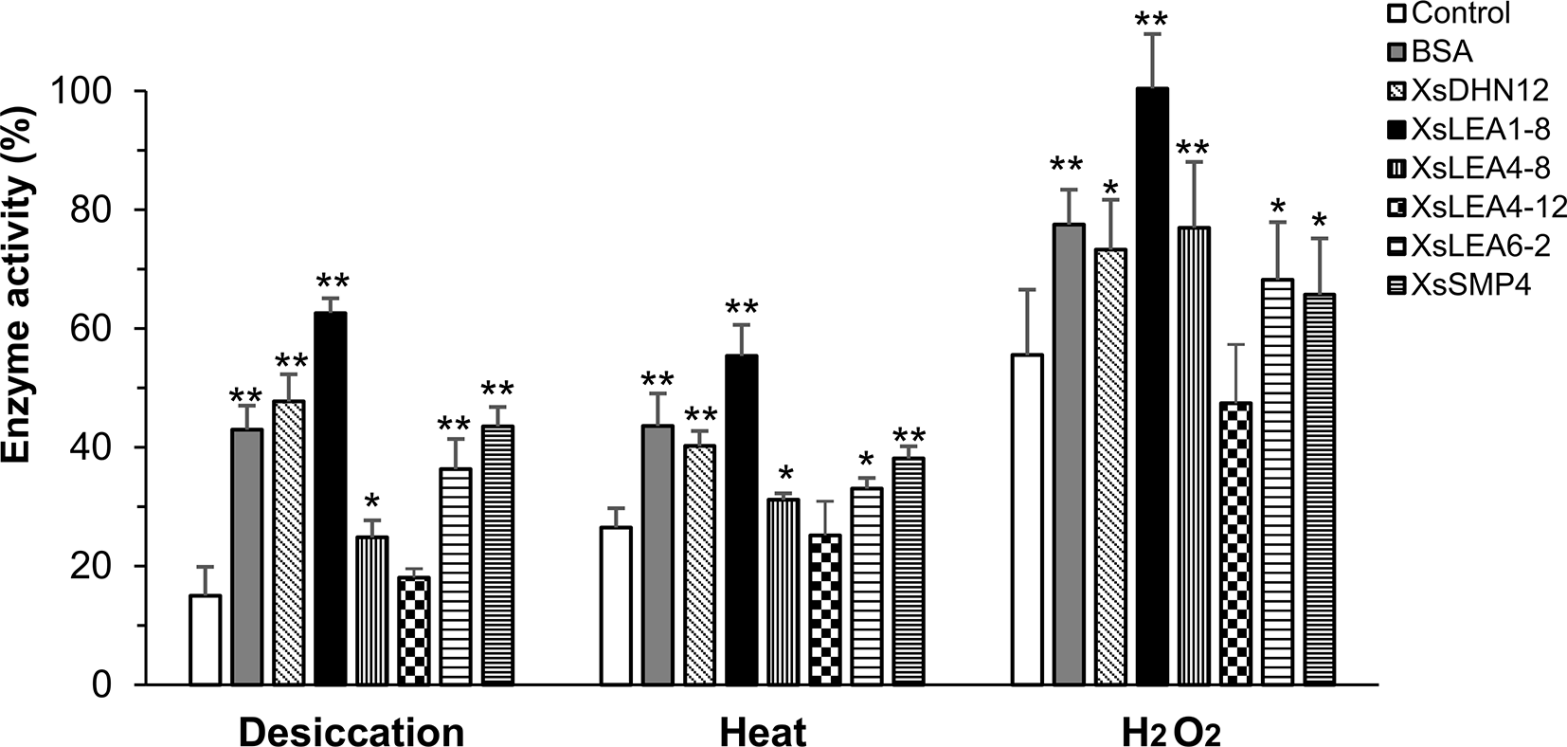
Protective function of *X. schlechteri LEAs* (XsLEAs) on lactate dehydrogenase (LDH) activity under stressful conditions. LDH by itself (negative control) or in the presence of one of the six purified XsLEAs or bovine serum albumin (BSA) (positive control) at a molar ratio of 1:1 were used to assess effects of desiccation, heat and oxidative stress. The experiments were repeated three times with three technical replicates in each experiment. Statistically significant differences as compared to control were analyzed using Student’s *t*-test (*p < 0.05 or **p < 0.01). The error-bars represent SD from nine replicates (*n* = 9).

We investigated the *in vitro* protective functions of the different XsLEAs on the activity of the enzyme LDH during desiccation, heat and oxidative stress (**Figure 2**).

Under desiccation, we observed a reduction of LDH activity to about 15% of control levels. With the exception of XsLEA4-12, all XsLEAs showed protective functionality against desiccation on the activity of LDH, with a protection of enzymatic activity up to 63% with XsLEA1-8. We found that heat stress reduced LDH activity to about 27% in the absence of XsLEAs, and XsLEA4-12 was unable to protect LDH activity. However, all other XsLEA proteins were able to protect LDH activity against heat, and again XsLEA1-8 showed the highest protective ability. The heat-protection activity of isolated LEA proteins has already been demonstrated in *in vitro* assays before (Goyal et al., 2005b; Kovacs et al., 2008; Halder et al., 2017), indicating that thermal anti-aggregation is a common feature of several LEA proteins.

The oxidative treatment imposed by H_2_O_2_ was less stressful as compared to desiccation and heat treatments. LDH activity dropped to 56% when treated with H_2_O_2_ and, with the exception of XsLEA4-12, all proteins tested were able to protect enzyme activity. Taken together, these results suggest that XsDHN12, XsLEA1-8, XsLEA4-8, XsLEA6-2, and XsSMP4 have chaperone-like activities for LDH activity upon desiccation, heat and oxidative stress, and that XsLEA4-12 is ineffective in the conditions herein tested.

### *In Vivo* Expression Of XsLEAs Enhances *E. coli* Viability Under Salt, Osmotic And Heat Stress

**Figure 3:**
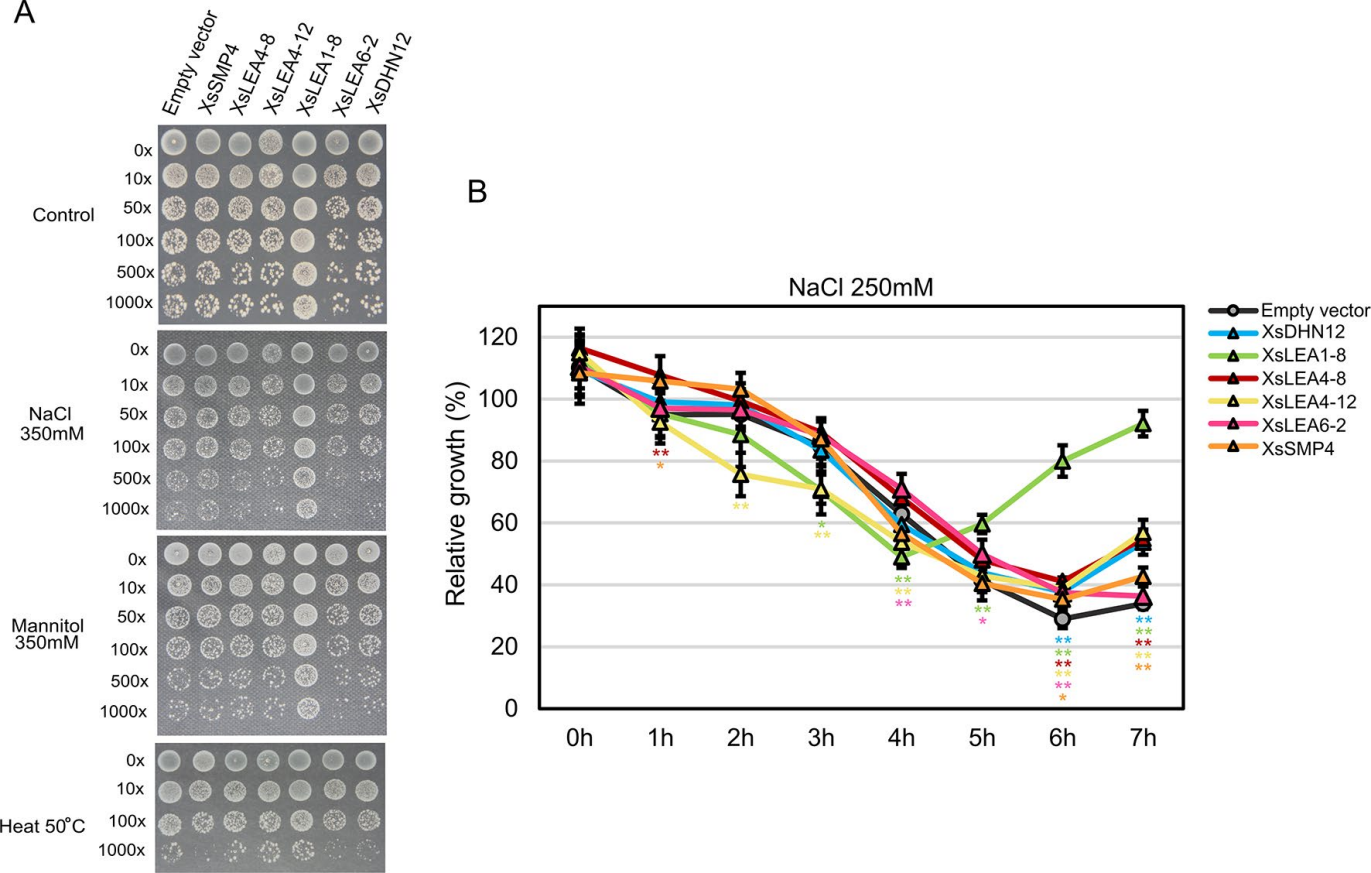
*In vivo* protective role of XsLEAs. **(A)** Response to salt, osmotic and heat stresses. **(B)** Relative growth of *E. coli* in liquid media with 250 mM NaCl. The experiments were repeated twice with three replicates per construct. Statistically significant differences were analyzed using Student’s *t*-test, and the bars indicates SD (*p < 0.05 or **p < 0.01).

To investigate effects of *XsLEA* expression on *E. coli* survival, we analyzed colony growth on plates with high concentrations of NaCl and mannitol, or when subjected to a heat shock (**Figure 3A**).

Under control conditions, the strain expressing *XsLEA6-2* displayed decreased growth than the empty vector-carrying strain, while the strain expressing *XsLEA1-8* displayed significant better growth than all other strains (data not shown). Interestingly, the *XsLEA1-8* strain also displayed better growth in all stressful conditions, suggesting that this protein has a higher protective function in *E. coli* when compared to the other tested XsLEAs. The strain with *XsLEA6-2* and *XsDHN12* presented a slower colony growth in all tested stresses, and, after heat treatment, the strain expressing *XsSMP4* also presented slower growth compared with the empty vector. These results show that not all XsLEAs have a protective function during *E. coli* growth under the tested conditions.

When analyzing relative growth of the strains in liquid media with a lower concentration of NaCl, we observed that after 6 h, all strains expressing *XsLEAs* displayed better growth than the empty vector (**Figure 3B**). Once more, the bacterial cells expressing *XsLEA1-8* showed a remarkable better growth recovery at the end of the experiment, indicating that this protein may perform a better protective function of *E. coli* upon salt stress as compared to the other tested XsLEAs. Interestingly, in the first 4 h of stress, a few strains displayed slower growth when compared to the empty vector. This observation might be due to leakiness of the expression vector, because the activation of the T7 promoter during bacterial growth prior induction will lead to production of recombinant protein which may limit bacterial growth (Grossman et al., 1998; Zhang et al., 2015; Briand et al., 2016). It is possible that the production of some XsLEAs during bacterial growth prior to the stress treatments might have detrimental effects on growth, likely due to unspecific binding to other molecules, furthermore, variations in the time and levels of protein produced in the different strains may interfere with the results observed. Taken together, our results point toward a potential protective role of XsLEA1-8 *in vivo*.

### Multiple Localizations of XsLEAs in Plant Cells

LEA proteins can be localized in various cell compartments, including the nucleus, cytosol, plasma membrane, mitochondria, plastids, endoplasmic reticulum (ER), and vacuoles (Hundertmark and Hincha, 2008; Candat et al., 2014). Prediction of subcellular localization indicates that XsDHN12, XsLEA1-8, XsLEA6-2, and XsSMP4 localize mainly in the nucleus and cytoplasm (**Supplementary Table 5**). The nuclear and cytoplasmic localization of proteins from families DHN, LEA_1, LEA_6, and SMP have been shown *in vivo* for *A. thaliana* (Candat et al., 2014), indicating that *in silico* analysis may have a strong correlation with *in vivo* analysis for these LEA families. XsLEA4-8 was predicted to be localized in the cytoplasm, nucleus, cell wall and plastids, and XsLEA4-12 in the nucleus, cytoplasm, and cell wall, corroborating the experimental data of multilocalization of *A. thaliana* LEA_4 proteins (Candat et al., 2014). Despite limitations of *in silico* predictions, these tools seem to be useful to design experiments for studying *in vivo* subcellular localization of LEA proteins.

To verify the *in vivo* subcellular localization of XsLEAs and to compare *in vivo* and *in silico* analysis, we expressed GFP fusions of XsLEA proteins in tobacco leaf epidermal cells under control of a 35S promotor (**Supplementary Figure 6**). The GFP-XsLEA proteins were localized throughout the cell compartments, especially in the cytoplasm, nucleus and membranes. GFP-XsLEA1-8, GFP-XsLEA4-8, and GFP-XsLEA6-2 accumulated mainly in the cytoplasm, membranes and nucleus, in the same way as the GFP empty vector. GFP-XsLEA4-12 showed a signal around the cells and in aggregate-like structures inside the cells which suggest that this protein may be secreted. GFP-XsSMP4 and GFP-XsDHN12 accumulated mainly in the membranes, and GFP-XsDHN12 also in the nucleus. In summary, our results indicate that the six *X. schlechteri* LEA proteins involved in DT in leaves are heterogeneously localized throughout various subcellular compartments. Further analyses combining C-terminal fusions and organelle specific markers are necessary to draw stronger conclusions on the specific localization of each of these proteins and further hypothesize about their cellular role.

### Heterologous Expression of XsLEA1-8 Enhances *A. thaliana* Stress Tolerance

*A. thaliana* ecotype Columbia-0 plants were transformed with a *35S::XsLEA1-8* construct, which led to a constitutive expression of the *XsLEA1-8* gene, including in the dry seed (**Supplementary Figure 7**). The phenotypic analysis of seeds expressing the *35S::XsLEA1-8* indicated that the germination percentages of WT and five independent lines did not differ significantly under control conditions (**Supplementary Figure 8**). During germination under salt, osmotic, and heat shock stress, a mild protective response was observed in two independent lines, indicating that XsLEA1-8 may not play a significant role in seed germination under the conditions tested here.

**Figure 4:**
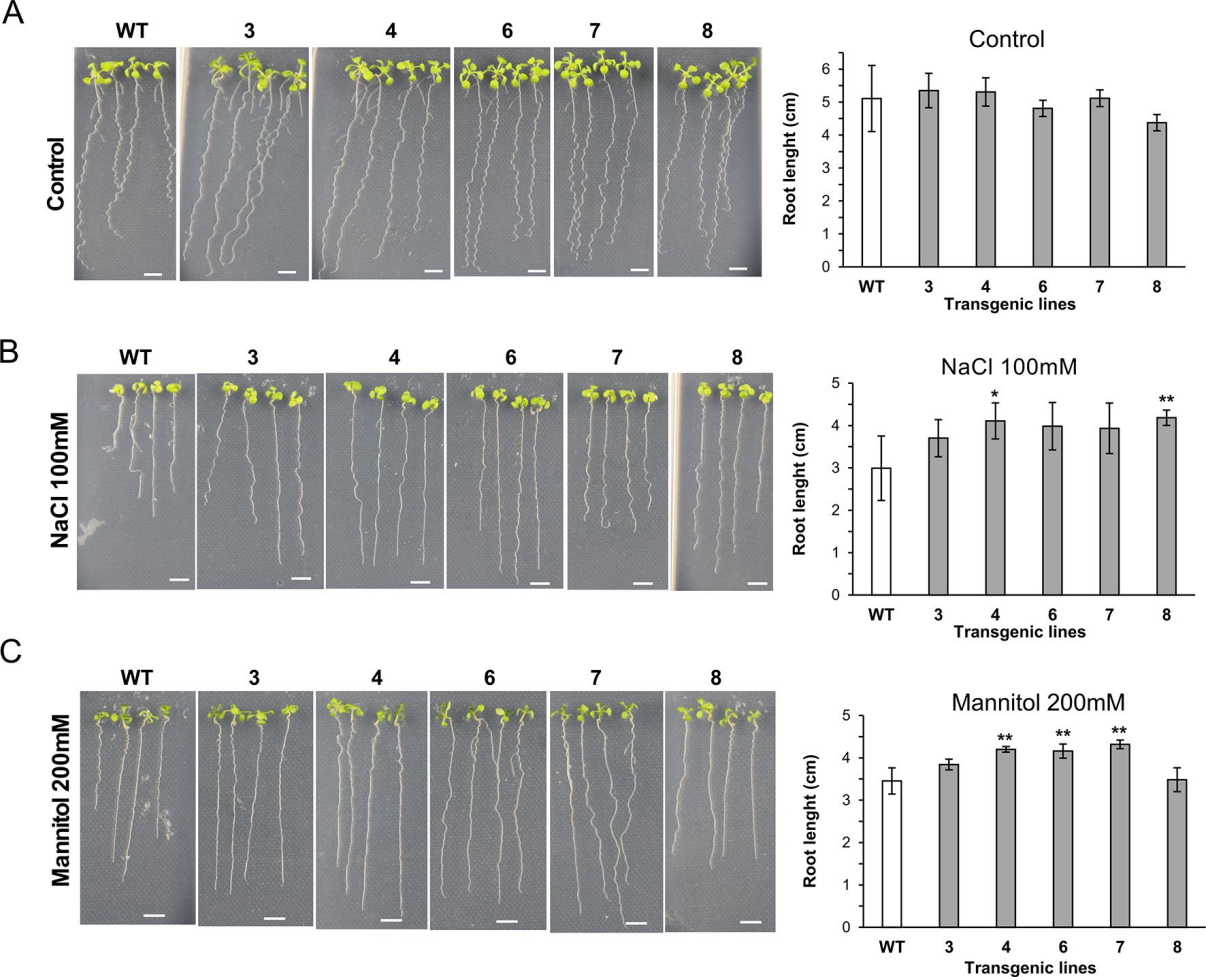
Phenotypic analysis of *A. thaliana* seedlings expressing *XsLEA1-8*. Seedlings grown on plates under control conditions **(A)**, 100 mM NaCl **(B)**, and 200 mM mannitol **(C)** are shown at the left. Data of one representative experiment are shown. Bars showing average root length ± SD (n = 3) are shown at the right. Statistically significant differences between the transgenic lines and the wild type (WT) were tested using Student’s *t*-test (*p < 0.05 or **p < 0.01). Scale bars = 0.5 cm.

We also investigated the growth ability of seedlings of *35S::XsLEA1-8* in media containing salt and mannitol (**Figure 4**). Under salt stress, the independent lines XsLEA1-8.4 and XsLEA1-8.8 displayed longer primary roots. Under osmotic stress imposed by mannitol, three independent lines (4, 6, and 7) displayed longer primary roots when compared with the wild-type. The phenotypic analysis of transgenic *A. thaliana* adult plants under drought stress showed that three independent lines (3, 7, and 8) displayed significantly higher relative water content at 12 days after withholding water (**Supplementary Figure 9A**); however, at this time point, no significant difference was observed in the DW of the transgenic lines when compared to the wild-type (**Supplementary Figure 9B**). These results suggest that the transgenic lines expressing *XsLEA1-8* may display a better control of water loss during drying, which may enhance their survival (**Supplementary Figure 9C**). Together, these findings point toward a potential role of XsLEA1-8 in enhancing osmotic stress tolerance in plants.

## Discussion

Since their discovery as accumulating during the later stages of seed embryogenesis, increasing evidence suggests a protective function of LEA proteins against desiccation and other stresses, leading to great interest in the structural dynamics of these proteins in the subcellular environment. As in seeds, LEA proteins have been shown to be an essential footprint of vegetative DT in resurrection plants. *X. schlechteri* is a monocot resurrection species whose genome has recently become available, enabling comparative genomic analysis with other monocots and resurrection species, as well as the understanding of the evolution and functional diversification of LEA proteins and their role in these organisms (Costa et al., 2017; Artur et al., 2018). *LEA* gene expression is upregulated in leaves of *X. schlechteri* between 60% and 40% RWC (1.5–1.0 gW/gDW) (**Supplementary Figure 1**), concomitant with the activation of the molecular signature of DT in seeds and resurrection plants (Illing et al., 2005; Leprince and Buitink, 2010; Costa et al., 2017).

*In silico* analysis of six *X. schlechteri* LEA proteins upregulated during desiccation indicated that these are typical IDPs, as they have IDRs in water, and properties, such as a high percentage of polar residues, low GRAVY scores, and extended disordered regions (Artur, 2019; Dunker et al., 2001; Tompa, 2002; Battaglia et al., 2008). Predictions of biochemical properties may give insight into the lack of structure of a protein. However, it is important to highlight that isolated parameters, such as length of polypeptide, net charge, or pI, cannot be used as a signature of unfolded or disordered structure (Uversky et al., 2000), revealing the necessity for more extensive *in silico* analysis to better predict the structural nature of such proteins. Using a specific predictor for Classification of Intrinsically Disordered Ensemble Regions (CIDER) (Holehouse et al., 2017), we found that XsLEAs belong to different IDP categories, such as environmentally determined disordered, globular, coiled, and chimeras of globular and coiled proteins. Two XsLEAs (XsSMP4 and XsLEA4-12) were predicted to contain structured regions, which is also a common feature of some IDPs able to form globules or chimeras of globules and coils and are able to undergo folding upon binding (Dyson and Wright, 2002; Dyson and Wright, 2005; Das et al., 2015). In fact, IDPs are able to interact with other molecules *via* Molecular Recognition Features (MoRFs) that undergo disorder-to-order transitions in the presence of binding partners (Mohan et al., 2006). We found that MoRFs are present in a low number within XsLEA sequences and, in general, they localize within predicted DBRs of the XsLEAs. MoRFs are thought to play important functions in protein–protein interactions related to signaling (Oldfield et al., 2005; Mohan et al., 2006; Disfani et al., 2012). In this way, the investigation of mutations of specific amino acids contributing to MoRFs will provide further insights into the specific role of these regions as well as interactions of LEA proteins with other proteins.

Reversible phosphorylation of IDPs is one of the major PTMs responsible for functional regulation of binding affinity of MoRFs (Iakoucheva et al., 2004; Mohan et al., 2006) and has already been shown to affect the function of LEA proteins in plants (Heyen et al., 2002; Alsheikh et al., 2003; Alsheikh et al., 2005; Brini et al., 2007; Liu et al., 2017). *In silico* analysis indicated that all the six XsLEAs have phosphorylation sites, with a remarkable high number in XsLEA4-12. However, in general, we found that the phosphorylation residues did not coincide with the predicted MoRF contributing residues, what suggests that phosphorylation may not play an important role in regulating binding affinity of MoRFs in the studied proteins.

In this study, we also investigated the *in vitro* folding dynamics of the six XsLEAs using circular dichroism (CD). Conformational changes of IDPs *in vitro* can be induced by changes in their environment such as pH, temperature, and presence of osmolytes or binding targets (Uversky, 2002; Uversky, 2009). Several studies have shown that LEA proteins are mainly disordered in aqueous solutions and are able to acquire secondary structures, mainly alpha-helices, upon desiccation and solute perturbations (Mouillon et al., 2006; Shih et al., 2010; Popova et al., 2011; Hundertmark et al., 2012; Shih et al., 2012; Cuevas-Velazquez et al., 2016). Our CD spectra corroborate the *in silico* predictions of the disordered (or unstructured) nature of the XsLEAs in aqueous solution (**Figure 1**). XsLEAs also possess residual structured regions which is also commonly found in IDPs (Tompa, 2012). Similar characteristics can be found in polypeptides containing local order, such as helix and beta-sheet like structures, and, in these proteins, secondary structure can be induced by variations in temperature, pH, presence of binding targets, osmolytes, and variable solvent concentrations (Shi et al., 2002; Soulages et al., 2002; Uversky, 2009; Rivera-Najera et al., 2014; Cuevas-Velazquez et al., 2016; Bremer et al., 2017). In fact, we observed that XsLEAs are able to acquire higher degrees of secondary structure in a hydrophobic solution of ACN. A highlight was the protein XsLEA1-8, which became fully alpha-helical in a solution of 80% ACN. ACN-mediated folding is triggered by significant reduction of hydration layers in direct contact with the protein surface, leading to conformational changes, such as formation of helices and sheets (Nelson et al., 1997; Gekko et al., 1998; Simon et al., 2001). This finding indicates that, under highly hydrophobic conditions, XsLEA proteins may acquire secondary structure, which may also be a factor regulating their functional activity.

It has already been shown that IDPs are able to preserve enzyme activity and avoid protein aggregation upon cellular stress by the ability to vitrify and trap cellular macromolecules into an amorphous matrix avoiding aggregation (Chakrabortee et al., 2007; Boothby et al., 2017). Several LEA proteins have been shown to protect enzymes from thermal and chemical inactivation and aggregation *in vitro* (Goyal et al., 2005a; Nakayama et al., 2007; Kovacs et al., 2008; Furuki and Sakurai, 2016; Liu et al., 2016; Agarwal et al., 2017; Halder et al., 2017). We investigated the ability to undergo conformational changes and acquire higher secondary structure under stress and the protective ability of XsLEAs. With the exception of XsLEA4-12, all studied proteins were able to preserve enzymatic activity upon desiccation, heat and oxidative stress *in vitro*, which supports the hypothesis that the high conformational changing ability correlates with protective abilities against aggregation and denaturation. It is important to highlight that since we undertook these experiments, new information on more reliable methods for IDPs quantification became available (Contreras Martos et al., 2018). As IDPs may show extreme variations in amino acid composition and physical properties, the use of more accurate quantification methods, such as ninhydrin and Qubit in combination with an absolute method, is recommended to draw more reliable conclusions about their structure. *In vivo* assays using *E. coli* have successfully demonstrated the protective role of LEA proteins upon stress (Zhang et al., 2014; Drira et al., 2015; Gao and Lan, 2016; Hu et al., 2016; Ling et al., 2016; Liu et al., 2016; Shi et al., 2016; Boothby et al., 2017; Jiang et al., 2017; Rakhra et al., 2017; Saucedo et al., 2017; Wang et al., 2017; Zhou et al., 2017). We found that several XsLEAs are able to improve bacterial growth under salt and osmotic stress, and the strains expressing XsLEA1-8 presented a faster stress recovery. Combined, these results indicate a correlation between *in vitro* and *in vivo* protective functions and point toward a potential application of the properties of XsLEA1-8 for engineering stability *in vitro* and *in vivo*.

Subcellular localization analysis assists in inferring function of proteins in plants. *A. thaliana* members of DHN, LEA_1, LEA_6, and SMP were shown to localize predominantly in the nucleus and cytoplasm, while LEA_4 members were multilocalized across chloroplasts, mitochondria, ER and pexophagosomes (Candat et al., 2014). *In silico* analysis of the six XsLEAs investigated in this study confirms the expected nucleo-cytoplasmic localization of XsDHN12, XsLEA1-8, XsLEA6-2, and XsSMP4, while XsLEA4-8 and XsLEA4-12 were predicted to also localize to the plastids and cell wall. In some cases, *in silico* and *in vivo* analysis may result in different results, as the example of an AdLEA protein from wild peanut, of which *in silico* analysis indicated localization mainly in the cytoplasm, while the GFP-fused protein was localized in the nucleus and cytoplasm (Sharma et al., 2016). In our *in vivo* analysis using GFP-fusions, subcellular locations of most XsLEAs were similar to the *in silico* predictions, showing that the latter can be useful for preliminary characterization of subcellular localization. To investigate the transferability of the protective properties shown by *in vitro* and *in vivo* assays in bacteria into enhancing plant stress tolerance, we developed transgenic *A. thaliana* plants constitutively expressing the *XsLEA1-8* gene. The expression of *XsLEA1-8* did not enhance stress tolerance during seed germination, but was able to enhance primary root growth under salt and osmotic stress in seedlings. Transgenic adult plants expressing *XsLEA1-8* displayed a higher absolute water content after 12 days of drought, indicating that this gene may play a role in plant drought tolerance.

Our study provides evidence for the structure–function relationship of LEA proteins expressed during desiccation in *X. schlechteri* plants. Our data reveal that *in silico* and *in vitro* analysis can provide useful information about LEA protein functions. We hypothesize that XsLEAs have been evolutionarily selected to be able to adopt diversified conformations driven by variations in their cellular environment. The conformational plasticity and multilocalization of XsLEAs may enable binding to essential cellular components (such as enzymes) and regulation of loss of water from cells, resulting in enhanced osmotic stress tolerance in *X. schlechteri* leaf cells. Furthermore, we believe that the high conformational plasticity and protective abilities of XsLEA1-8 make it a potential candidate for engineering biostability *in vitro* by serving as a model for synthetic chaperons, as well as for enhancing drought tolerance in crop species.

## Data Availability Statement

All datasets generated for this study are included in the manuscript/**Supplementary Files**.

## Author Contributions

MA performed the *in silico* analysis and data interpretation. MA and JR performed gene cloning for bacterial assays. JR performedgene cloning for plant heterologous expression. MA: MA performed the *in vitro* assays and data analysis with significant contribution of TD. HH, WL and JF participated in the design of the study and supervised the project. MA wrote the manuscript. All authors contributed with comments and revision of the manuscript.

## Funding

MA received financial support from CAPES-Brazilian Federal Agency for Support and Evaluation of Graduate Education (BEX 0857/14-9). Conformation analyses and enzyme protection assays were funded by a DST-NRF South African Research Chair grant (number 98406) awarded to JF.

## Conflict of Interest

The authors declare that the research was conducted in the absence of any commercial or financial relationships that could be construed as a potential conflict of interest.

## Footnotes

1 *The Xerophyta viscosa (Baker) plants used by* Costa et al. (2017) *have been classified as Xerophyta schlechteri (Baker) N.L. Meneses* (Behnke et al., 2013).
