# Supplementary material for: Structural Plasticity of Intrinsically Disordered LEA Proteins from Xerophyta schlechteri Provides Protection In Vitro and In Vivo
Source: Front Plant Sci. 2019 Oct 10;10:1272. doi: 10.3389/fpls.2019.01272 (PMC6798065; doi:10.3389/fpls.2019.01272)
Supplement: Supplementary file 1 [file DataSheet_1.pdf]

Supplementary Figures

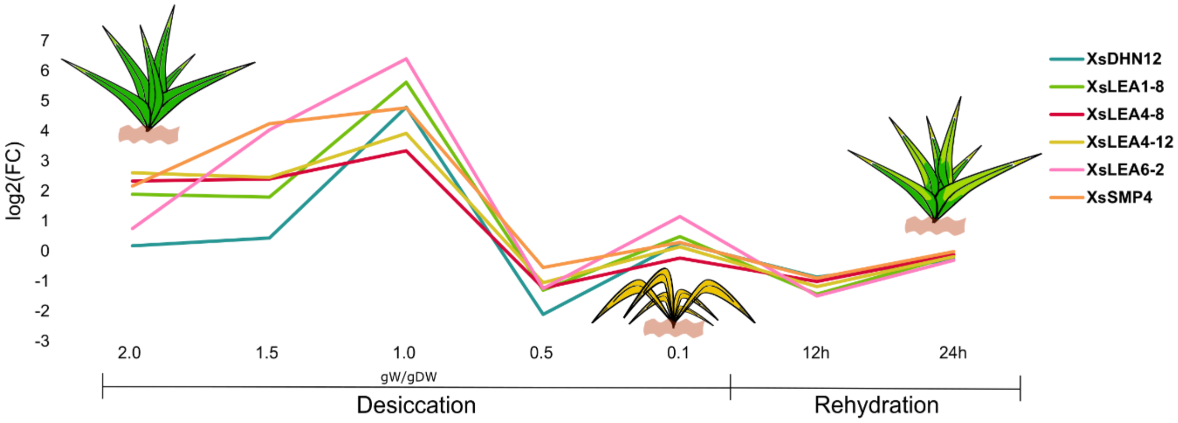

Supplementary figure 1. Expression patterns of *X. schlechteri LEAs*. The log2fold change was calculated relative to the expression in the previous time-point. Data from Costa et al. (2017).

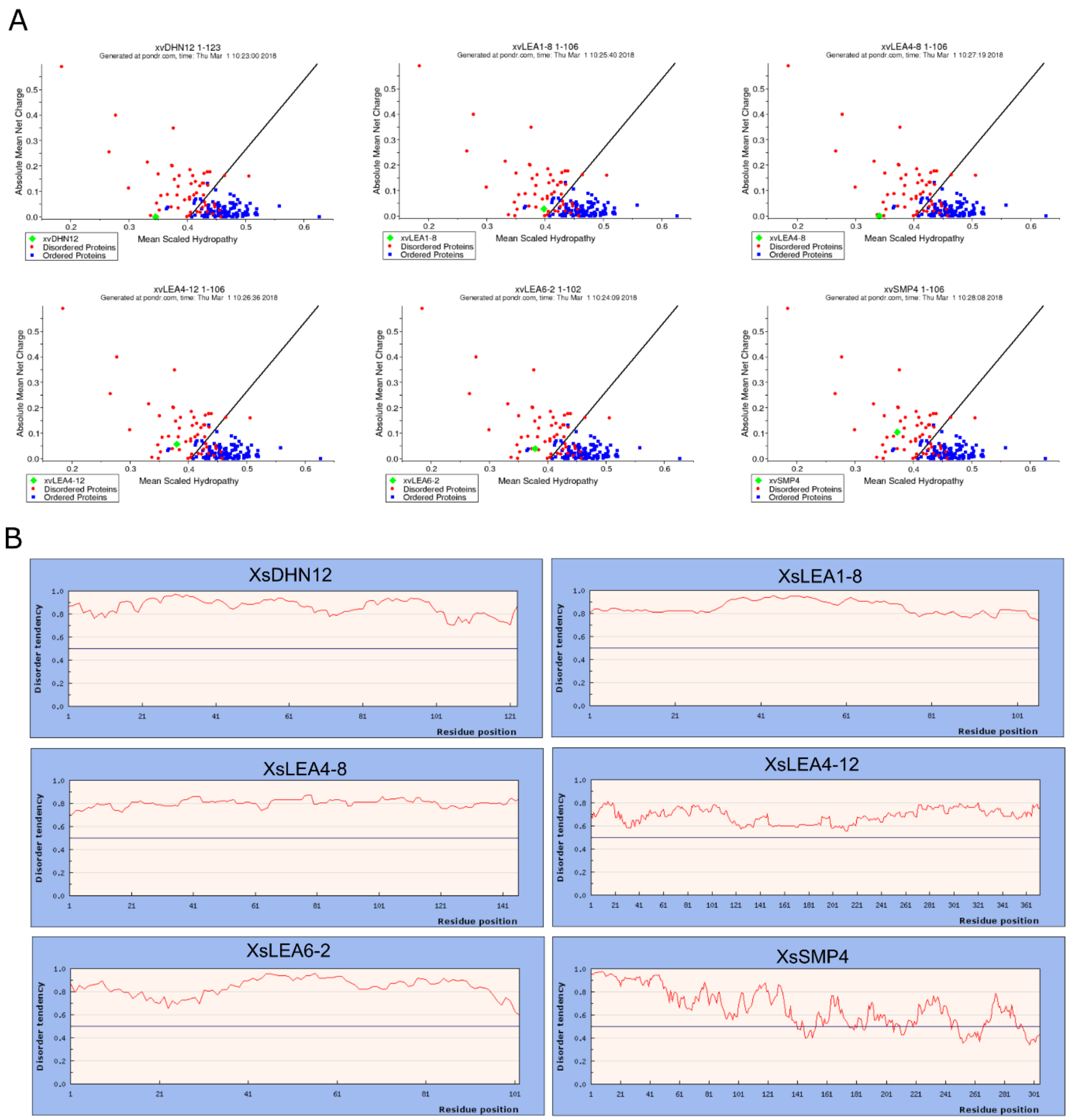

Supplementary figure 2. *In silico* predictions of intrinsic disorderedness of six XsLEAs. **(A)** Charge-hydropathy (C-H) plot including LEAs from *X. schlechteri* (green diamonds) that are plotted together with known globular proteins (blue squares) and known intrinsically disordered proteins (red circles). The prediction was performed with PONDR (http://www.pondr.com/). **(B)** Disorder tendency of the six XsLEAs predicted with IUPRED (Dosztanyi et al., 2005). Residues above 0.5 are predicted to be disordered, while those below 0.5 are considered ordered.

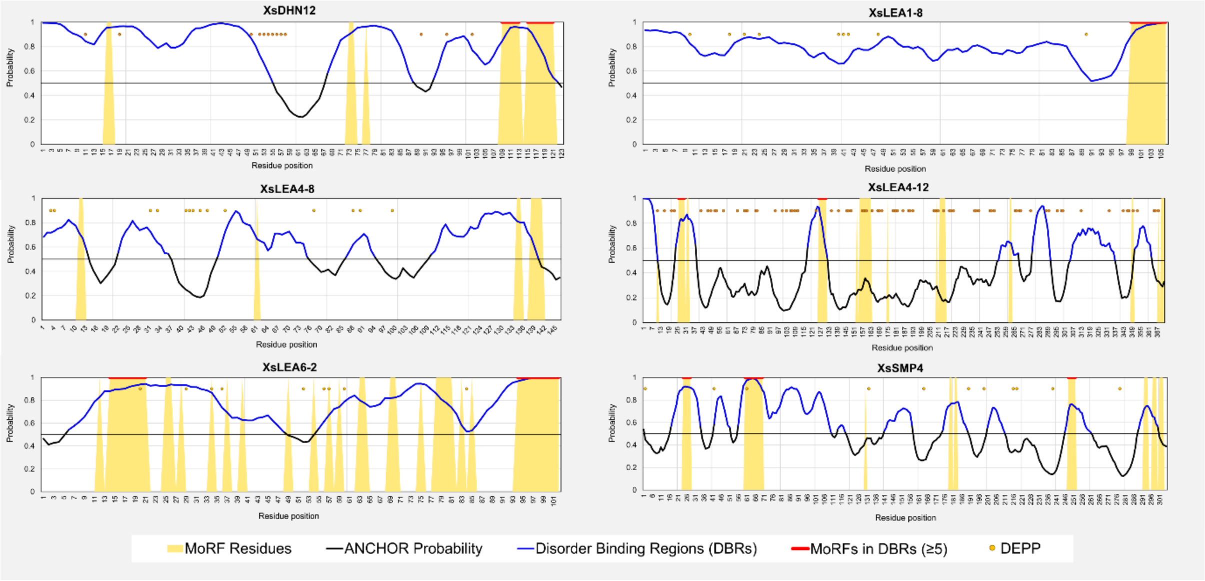

Supplementary figure 3. *In silico* prediction of intrinsic disorder properties of six XsLEAs. The plots show the ANCHOR probability of disorder binding regions (line), the positions of Molecular Recognition Feature (MoRF) residues (yellow blocks), and the disordered binding regions (DBRs) with a probability above 0.5 of the ANCHOR probability (blue highlighted line). Positions containing more than 5 MoRF residues within DBRs are indicated by a red line and phosphorylation prediction of residues (Y,S,T) is shown by orange dots.

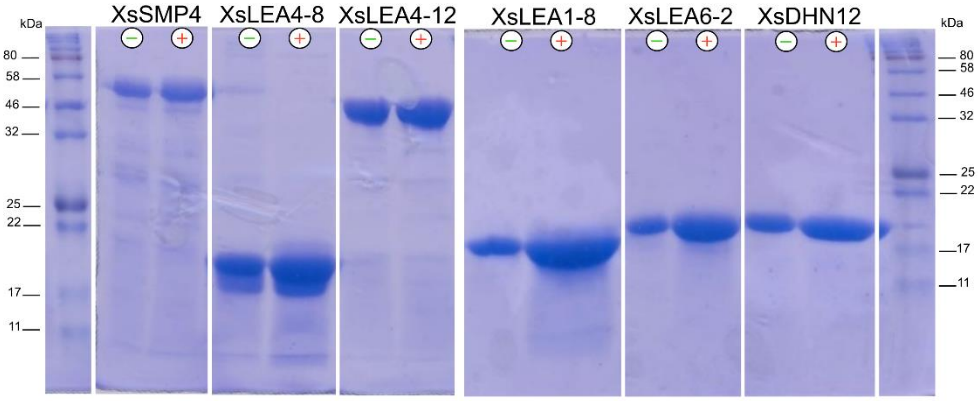

Supplementary figure 4. Coomassie stained 12% SDS-PAGE gel showing purified recombinant XsLEA proteins. (-) Indicates 10µg of purified recombinant proteins loaded on gel without heating and (+) indicates 15µg of purified recombinant proteins loaded on gel after an extra heating step at 97°C for 10 minutes.

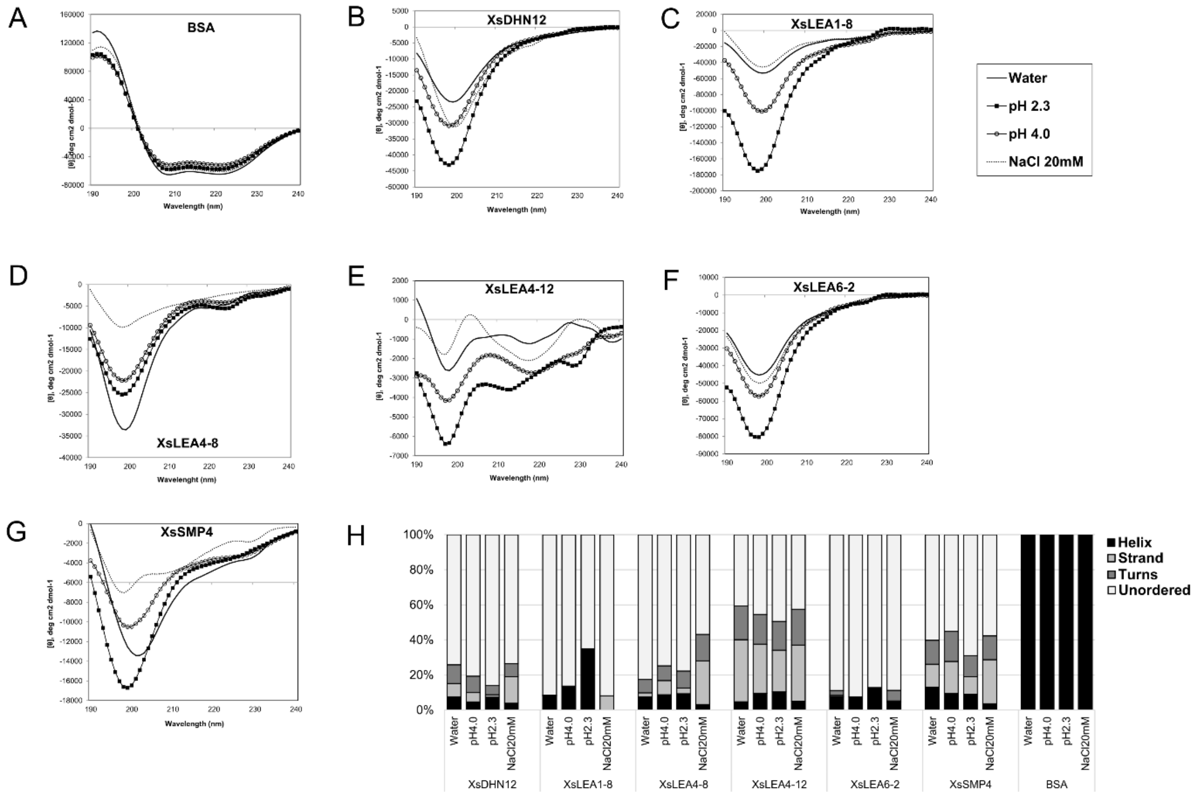

Supplementary figure 5. Circular dichroism analysis of XsLEAs. CD spectra of BSA and *X. schlechteri* LEA proteins **(A-G)**. The CD spectra was obtained in water, water adjusted with 1M HCl to pH 2.3 and pH 4.0, and in 20mM NaCl. All the spectra were analysed at room temperature. The solutions were prepared about 1 hour before acquisition of the spectra. The graphs show the spectra obtained after subtracting the reads of a blank sample (water only). **(H)** Secondary structure content. Predictions of the content of helix, strand, turns and unordered regions for the different proteins were performed with Dichroweb.

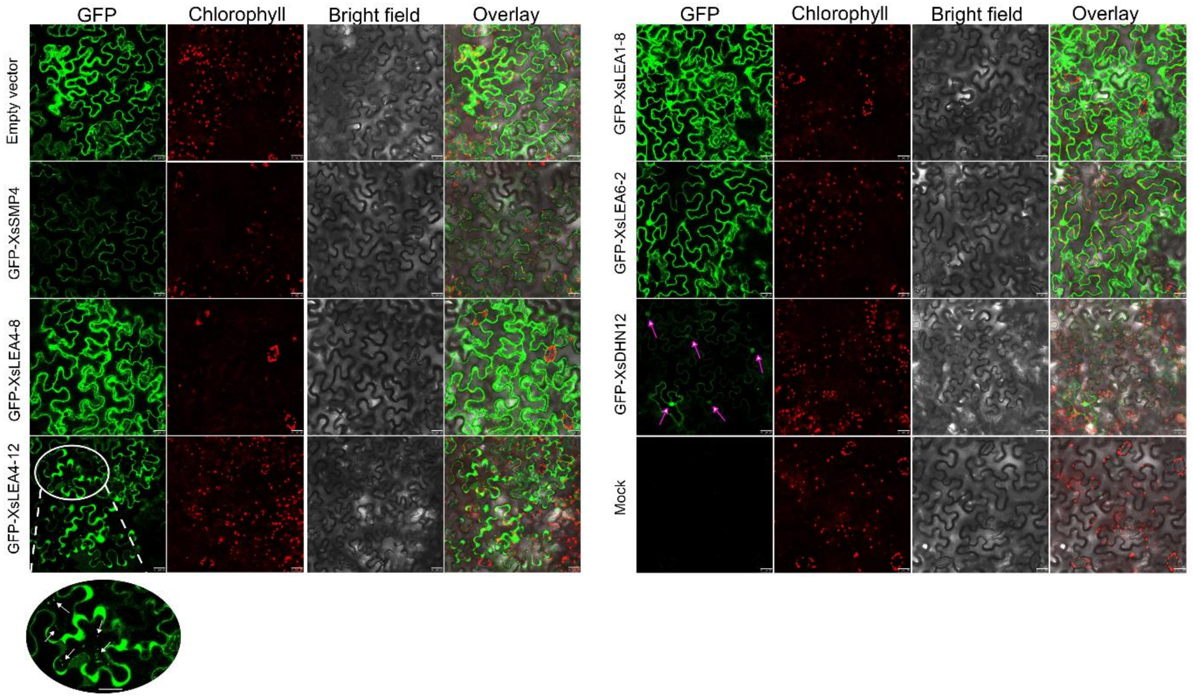

Supplementary figure 6. Subcellular localization of the GFP fusion proteins in transgenic *N.*

benthamiana plants. The subcellular distribution of the GFP fusions and control GFP in the epidermal leave cells of transgenic tobacco leaves were visualized using laser confocal scanning microscopy. Red indicates the auto fluorescence of chlorophyll and GFP fluorescence is presented in green. Pink arrows in GFP-XsDHN12 indicate the nucleus. The white arrows in the circle snapshot of GFP-XsLEA4-12 show vesicle-like structures. Scale bars = 25µM.

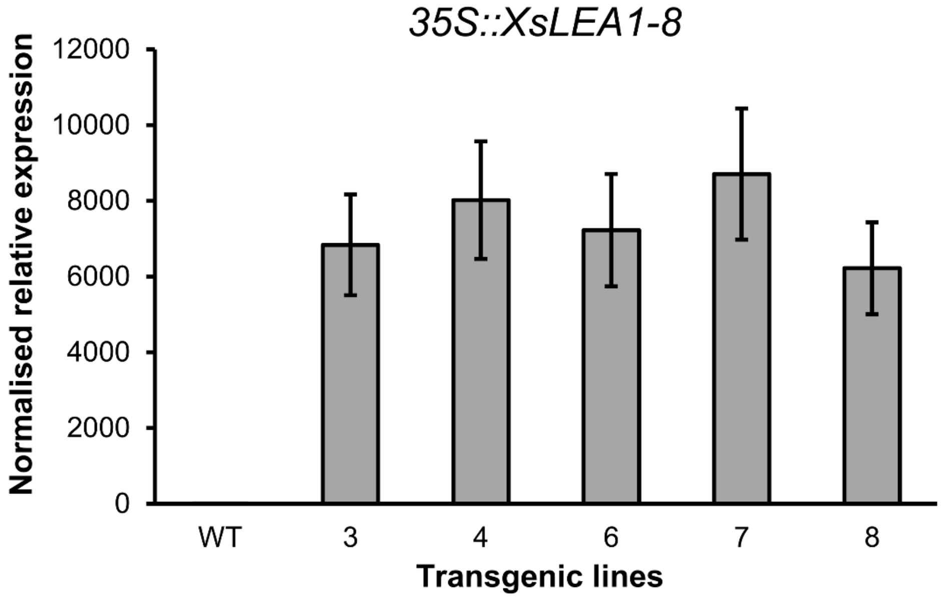

Supplementary figure 7. Expression of 35S::*XsLEA1-8* in *A. thaliana*. RT-qPCR analysis was performed in cDNA produced from RNA extracted from *A. thaliana* dry seeds in order to confirm the expression of 35S::*XsLEA1-8*. Data was analysed according to the ΔΔCT method. Bars indicate means ± SD (*n* = 3).

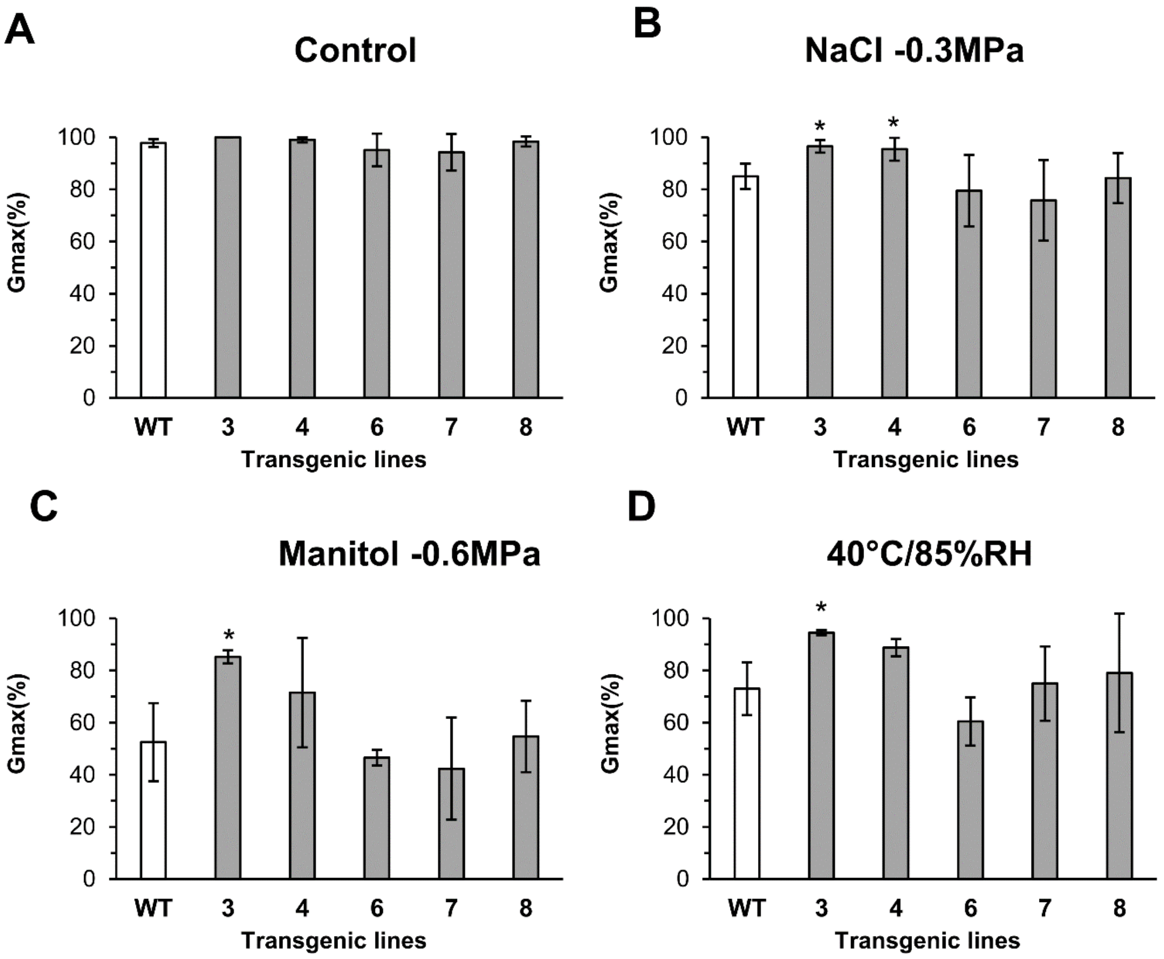

Supplementary figure 8. Germination of transgenic 35S:*XsLEA1-8 A. thaliana* seeds under stress. **(A)** Seeds were stratified at 4°C for 48 hours and germinated for 5 days at 22°C and continuous light. **(B)** Stratification and germination in the presence -0.3MPa of NaCl. **(C)** Stratification and germination in the presence -0.6MPa of Mannitol. **(D)** Heat stress at 40°C and 85% relative humidity (RH) prior to germination at 22°C and continuous light. Statistically significant differences were analysed using Student's *t*-test (* $p<0.05$).

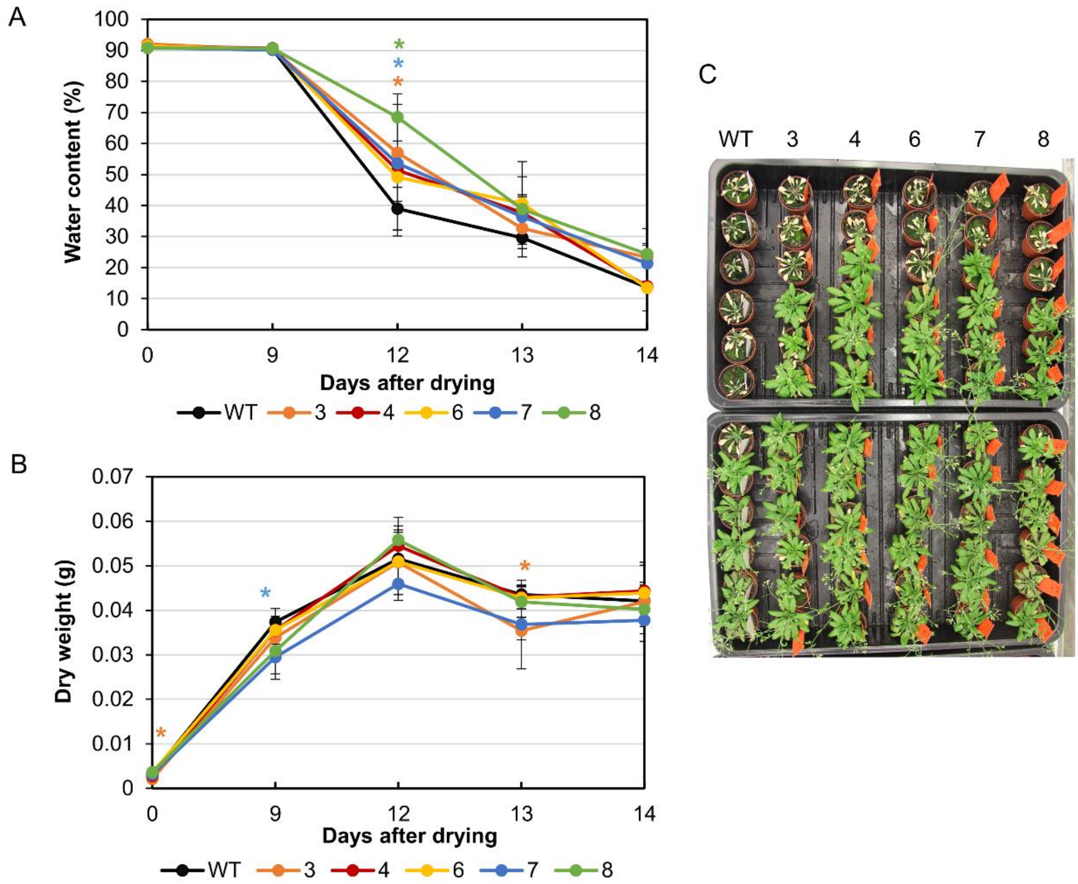

Supplementary figure 9. Phenotypic analysis of *A. thaliana* adult plants expressing 35S::*XsLEA1-8* under drought. **(A)** Relative water content and **(B)** dry weight of adult plants well-irrigated (day 0), or after withholding water for 9, 12, 13 and 14 days. Statistically significant differences between transgenic independent lines (3, 4, 6, 7, and 8) and the wild-type (WT) were analysed using Student's *t*-test. Error bars indicate means ± SD (* $p<0.05$). **(C)** Plants rehydrated for 7 days after 12 days of drought.
